# Electrophysiological characteristics and ablation of ventricular arrhythmias originating from the intramural basal inferior septum

**DOI:** 10.1093/europace/euae001

**Published:** 2024-01-05

**Authors:** Jie Yang, Mengmeng Li, Chenxi Jiang, Ribo Tang, Caihua Sang, Wei Wang, Xin Zhao, Changyi Li, Songnan Li, Xueyuan Guo, Changqi Jia, Man Ning, Li Feng, Dan Wen, Hui Zhu, Yuexin Jiang, Tong Liu, Fang Liu, Deyong Long, Jianzeng Dong, Changsheng Ma

**Affiliations:** Department of Cardiology, Beijing Anzhen Hospital, Capital Medical University, National Clinical Research Center for Cardiovascular Diseases, No. 2, Anzhen Road, Chaoyang District, Beijing 100029, China; Department of Cardiology, Beijing Anzhen Hospital, Capital Medical University, National Clinical Research Center for Cardiovascular Diseases, No. 2, Anzhen Road, Chaoyang District, Beijing 100029, China; Department of Cardiology, Beijing Anzhen Hospital, Capital Medical University, National Clinical Research Center for Cardiovascular Diseases, No. 2, Anzhen Road, Chaoyang District, Beijing 100029, China; Department of Cardiology, Beijing Anzhen Hospital, Capital Medical University, National Clinical Research Center for Cardiovascular Diseases, No. 2, Anzhen Road, Chaoyang District, Beijing 100029, China; Department of Cardiology, Beijing Anzhen Hospital, Capital Medical University, National Clinical Research Center for Cardiovascular Diseases, No. 2, Anzhen Road, Chaoyang District, Beijing 100029, China; Department of Cardiology, Beijing Anzhen Hospital, Capital Medical University, National Clinical Research Center for Cardiovascular Diseases, No. 2, Anzhen Road, Chaoyang District, Beijing 100029, China; Department of Cardiology, Beijing Anzhen Hospital, Capital Medical University, National Clinical Research Center for Cardiovascular Diseases, No. 2, Anzhen Road, Chaoyang District, Beijing 100029, China; Department of Cardiology, Beijing Anzhen Hospital, Capital Medical University, National Clinical Research Center for Cardiovascular Diseases, No. 2, Anzhen Road, Chaoyang District, Beijing 100029, China; Department of Cardiology, Beijing Anzhen Hospital, Capital Medical University, National Clinical Research Center for Cardiovascular Diseases, No. 2, Anzhen Road, Chaoyang District, Beijing 100029, China; Department of Cardiology, Beijing Anzhen Hospital, Capital Medical University, National Clinical Research Center for Cardiovascular Diseases, No. 2, Anzhen Road, Chaoyang District, Beijing 100029, China; Department of Cardiology, Beijing Anzhen Hospital, Capital Medical University, National Clinical Research Center for Cardiovascular Diseases, No. 2, Anzhen Road, Chaoyang District, Beijing 100029, China; Department of Cardiology, Beijing Anzhen Hospital, Capital Medical University, National Clinical Research Center for Cardiovascular Diseases, No. 2, Anzhen Road, Chaoyang District, Beijing 100029, China; Department of Cardiology, Beijing Anzhen Hospital, Capital Medical University, National Clinical Research Center for Cardiovascular Diseases, No. 2, Anzhen Road, Chaoyang District, Beijing 100029, China; Department of Cardiology, Beijing Anzhen Hospital, Capital Medical University, National Clinical Research Center for Cardiovascular Diseases, No. 2, Anzhen Road, Chaoyang District, Beijing 100029, China; Department of Cardiology, Beijing Anzhen Hospital, Capital Medical University, National Clinical Research Center for Cardiovascular Diseases, No. 2, Anzhen Road, Chaoyang District, Beijing 100029, China; Department of Cardiology, Beijing Anzhen Hospital, Capital Medical University, National Clinical Research Center for Cardiovascular Diseases, No. 2, Anzhen Road, Chaoyang District, Beijing 100029, China; Department of Cardiology, Beijing Anzhen Hospital, Capital Medical University, National Clinical Research Center for Cardiovascular Diseases, No. 2, Anzhen Road, Chaoyang District, Beijing 100029, China; Department of Cardiology, Beijing Anzhen Hospital, Capital Medical University, National Clinical Research Center for Cardiovascular Diseases, No. 2, Anzhen Road, Chaoyang District, Beijing 100029, China; Department of Cardiology, Beijing Anzhen Hospital, Capital Medical University, National Clinical Research Center for Cardiovascular Diseases, No. 2, Anzhen Road, Chaoyang District, Beijing 100029, China; Department of Cardiology, Beijing Anzhen Hospital, Capital Medical University, National Clinical Research Center for Cardiovascular Diseases, No. 2, Anzhen Road, Chaoyang District, Beijing 100029, China; Department of Cardiology, Beijing Anzhen Hospital, Capital Medical University, National Clinical Research Center for Cardiovascular Diseases, No. 2, Anzhen Road, Chaoyang District, Beijing 100029, China

**Keywords:** Catheter ablation, Ventricular arrhythmias, Preferential activation exit, Basal inferior septum, Activation mapping, Pacing mapping

## Abstract

**Aims:**

The electrocardiographic and electrophysiological characteristics of ventricular arrhythmia (VA) arising from the intramural basal inferior septum (BIS) have not been specifically addressed to date. The aim of the current study was to characterize intramural BIS-VA and distinguish it from those with endocardial origins besides clarifying the anatomical configurations of the pyramidal space.

**Methods and results:**

Fifty-five consecutive patients undergoing catheter ablation of VAs from BIS were identified and divided into three groups: the left ventricular (LV)-BIS group (*n* = 28), right ventricular (RV)-BIS group (*n* = 8), and intramural group (Intra, *n* = 19). Compared with the LV-BIS and RV-BIS groups, patients in the Intra group presented with no adequate earliest activation time at the two-sided BIS and epicardial coronary system [right: 7.79 ± 2.38 vs. left: 7.16 ± 2.59 vs. the middle cardiac vein (MCV): 6.26 ± 1.73 ms, *P* = 0.173] and poor-matched pacing-produced QRS at each site. Under the intracardiac echocardiography view, the pyramidal base was the broadest part of the septum and served as the division of the two-sided BIS. Focal ablation yielded promising acute-term and long-term procedural success in the LV-BIS and RV-BIS groups. But for the Intra group, VAs disappeared only after stepwise ablation successively targeted early preferential exit. After follow-up, three patients in the Intra group had recurrent VA, and all of them were treated well by a redo procedure or drug therapy.

**Conclusion:**

Intramural VAs were relatively common in the BIS region in our series. Intra-procedural mapping was important to distinguish the intramural VAs from other VAs by comparing the local activation time and pacing mapping. Procedural success could be achieved by stepwise ablation on the counterpart sides of the BIS and within the MCV.

## Graphical Abstract

**Graphical Abstract:**
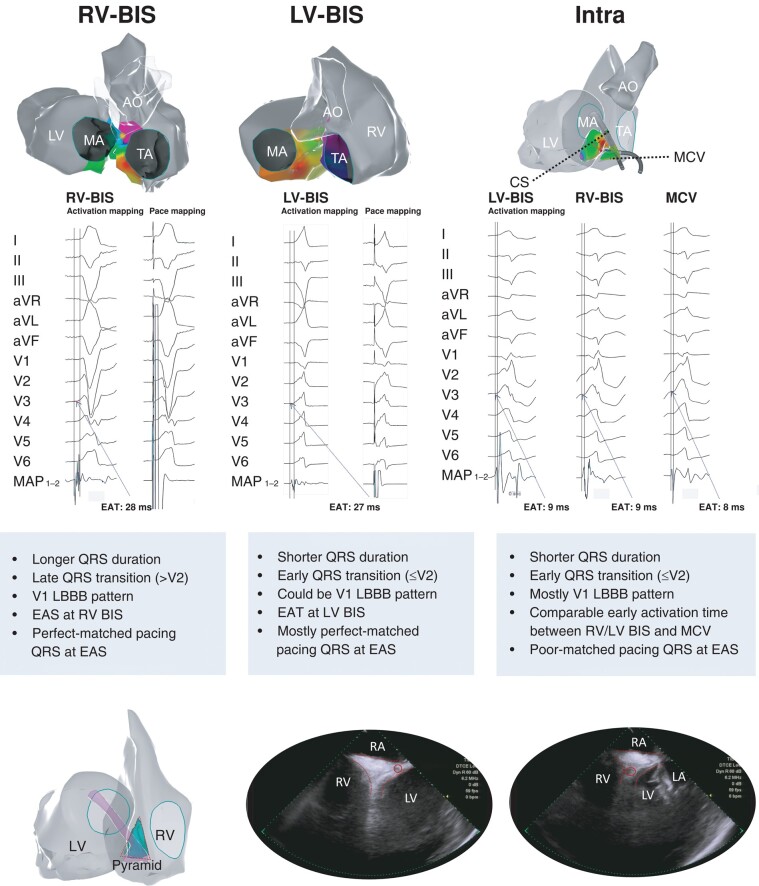

**What’s new?**

- Intramural ventricular arrhythmia (VA) was relatively common in the basal inferior septum (BIS) region in our series, and the pyramidal base was the broadest part of the septum and served as the division of the two-sided BIS.
- A scalar electrocardiogram provided limited clues to intramural VA, while intracardiac mapping was important to distinguish intramural foci from other VAs.
- Intramural BIS-VA was typically characterized by a nearly simultaneous activation time recorded in the two-sided BIS as well as the middle cardiac vein (MCV), and poor-matched pacing QRS morphology to clinical VA.
- A stepwise ‘kissing’ ablation subsequently targeting preferential exit at the two-sided BIS and MCV was required to eliminate intramural BIS-VA.
- Special attention should be paid to atrial-ventricular conduction during ablation.

## Introduction

Catheter ablation of ventricular arrhythmia (VA) remains challenging, especially for those with intramural or epicardial origins.^1^ The basal inferior septum (BIS), surrounded by the mitral annulus (MA), tricuspid annulus (TA), and interventricular groove, is an important but under-recognized source of VA. VA arising from the BIS varies from the endocardium, the intramural to the epicardium, and usually poses a great challenge to clinical diagnosis and ablation. Some previous studies have reported the electrocardiographic features and ablation outcomes of endocardial and epicardial BIS-VAs.^2–5^ However, it remains unclear how electrocardiogram (ECG) and intracardiac mapping could be integrated to optimize the ablation strategy, especially for those with intramural origins. Furthermore, the BIS itself is a component of the inferior pyramidal space, a ‘sandwich’ structure with a close apposition of atrial and ventricular musculature. Its anatomical relevance to intramural BIS-VA has not yet been determined on *in vivo* patients. By retrospectively reviewing a cohort of patients with BIS-VAs, the present study aims to clarify the clinical and electrophysiological characteristics of BIS-VAs with intramural origins and characterize the anatomical configurations of the BIS and its adjacent structures.

## Methods

### Study population

Between January 2017 and January 2022, consecutive patients with VAs originating from the BIS were screened, and those in whom the intramural origin was identified were included in this study. The comparison cohort included patients with VAs originating from the endocardial aspect of the BIS during the same period. The procedural data were retrospectively reviewed and analysed by two independent electrophysiologists. Patients with significant structural heart diseases revealed by transthoracic echocardiography or magnetic resonance were excluded from the analysis. This study complied with the Declaration of Helsinki, and the protocol was approved by institutional review boards at Beijing Anzhen Hospital. All patients provided written informed consent prior to the procedure.

### Mapping strategy and ablation procedure

All antiarrhythmic drugs were withdrawn for at least five half-lives before the commencement of procedures (4 weeks longer for amiodarone users). The procedures were performed under local anaesthesia and conscious sedation. A quadripolar catheter was positioned at the right ventricular (RV) apex to induce ventricular tachycardia (VT). In patients with no frequent premature ventricular contraction (PVC) or sustained VT at the baseline or after ventricular stimulation, intravenous isoproterenol (0.5–2.0 µg/min) was administrated. A 3.5 mm, deflectable quadripolar saline-irrigated catheter (ThermoCool SmartTouch, Biosense Webster Inc., Diamond Bar, CA, USA) compatible with the three-dimensional (3D) electroanatomic mapping system (Carto 3, Biosense Webster Inc.) was used for mapping and ablation. A 10 Fr intracardiac echocardiography (ICE) probe (SoundStar, Biosense Webster Inc.) was routinely used to display the left ventricular (LV) and RV anatomy and assist mapping and ablation in our centre. The entire RV and LV were reconstructed by rotating the ICE probe within the right atrium and RV with no <20 sectors in each chamber. The TA and MA were tagged during reconstruction. The geometry of the pyramidal space was reconstructed offline after the index procedure by reviewing the ICE sectors from both ventricles. The height and thickness of the pyramidal space were specifically measured by ICE.

Initial activation mapping was performed either from the LV or the RV at the discretion of the operators. Before ablation, bi-ventricular activation mapping was performed in all patients. The epicardial venous system, including the middle cardiac vein (MCV), was mapped if no adequate earliest activation site (EAS) was recorded on the LV and RV endocardial surfaces. Pace mapping at the respective EASs was also performed in all patients with an initial output of 5 mA at 2 ms pulse width, and this increased to 10 mA if the local myocardium was not captured. Pace mapping was considered perfectly matched when the QRS complex was identical in 12 leads.

The VA was considered as the LV (LV-BIS) or RV (RV-BIS) local superficial origin when: (i) there is a preferential exit (namely the EASs of all mapping sites) with a local activation time of at least 20 ms earlier than that on the opposite side; (ii) local pacing produced a QRS identical to clinical VA, and (iii) endocardial ablation terminated the VA within 5 s. The VA was considered to be of intramural origin (the Intra group) when: (i) the earliest activation time (EAT) on either aspect of the BIS was nearly simultaneous with the local activation time difference of <10 ms^6^ and (ii) the pace-produced QRS morphology at the exits on both sides was poorly matched with the clinical VAs.^1^

Upon identification of the EAS on either side of the BIS, radiofrequency ablation was attempted with an energy output of 35–40 W with an irrigated saline of 17 mL/min and a contact force of above 7 g. If VA suppression occurred during ablation, another 40–60 s of ablation was delivered at the same site. Otherwise, subsequent mapping and ablation were switched to the opposite side of the BIS. Ablation within the MCV was performed after failure in endocardial ablation. Energy delivery in the MCV started at 25 W and was titrated up to 30 W with saline irrigation at 25 mL/min. The procedural endpoints were no spontaneous VAs during a 30 min waiting time and non-inducibility of VAs by rapid ventricular pacing combined with isoproterenol administration. Atrial-ventricular conduction was closely monitored during ablation.

### Follow-up

All patients off antiarrhythmic drugs were followed up for ≥12 months. The follow-up included a 12-lead ECG and 24 h ambulatory ECG, outpatient visits, and telephone interviews every 3 months after the index procedure or whenever symptoms associated with VA occurred. Long-term ablation success was defined as ≥80% reduction in PVC burden through Holter monitoring or no recurrent VT during follow-up.

### Statistical analysis

For categorical variables, data were expressed as frequencies and percentages. Continuous variables were expressed as means ± standard deviations. Continuous data were compared with Student’s *t*-test or analysis of variance. Categorical variables were compared using the *χ*^2^ or Fisher exact test, as appropriate. A *P*-value of <0.05 was considered statistically significant. All statistical analyses were performed with SPSS 19.0 (SPSS Inc., Chicago, IL, USA).

## Results

### Clinical characteristics

Among 2502 patients receiving catheter ablation for VA in our centre during the study period, 55 (2.20%) patients (average age of 52.55 ± 13.36 years old, female/male = 8/47) in whom BIS origins were identified were included in the study. Forty-seven (85.45%) of these patients presented with frequent PVC and eight patients (14.55%), with monomorphic VT. All patients had a structurally normal heart with an LV ejection fraction of 62.62 ± 5.83% and an LV end-diastolic diameter of 49.82 ± 4.64 mm. The baseline PVC burden was 25.77 ± 5.47%. After isoproterenol infusion or ventricular stimulation, VAs were consistently presented during the whole procedure.

Based on the VA origins, the patients were divided into three groups: the LV-BIS group (*n* = 28), the RV-BIS group (*n* = 8) group, and the intramural origin group (Intra, *n* = 19). The clinical characteristics are summarized in *Table 1*. No significant differences in clinical characteristics were observed among the three groups, except with regard to the younger age of patients in the RV-BIS group.

**Table 1:** Patients’ characteristics (*N* = 55)

	LV-BIS (*N* = 28)	RV-BIS (*N* = 8)	Intra (*N* = 19)	*P*-value
Age, years	56.96 ± 11.46	41.38 ± 14.99	50.74 ± 12.72	0.012
Sex, male *n* (%)	23 (82.14)	6 (75.00)	18 (94.74)	0.322
LVEF, %	62.44 ± 4.26	65.75 ± 7.81	61.83 ± 7.25	0.513
LVEDD, mm	49.28 ± 4.84	48.71 ± 4.27	51.25 ± 4.60	0.412
History of VT, *n* (%)	2 (7.14)	1 (12.50)	5 (26.32)	0.185
Previous ablation, *n* (%)	5 (17.86)	0	3 (15.79)	0.442

BIS, basal inferior septum; Intra, intramural; LV, left ventricle; LVEDD, left ventricular end-diastolic diameter; LVEF, left ventricular ejection fraction; RV, right ventricle; VT, ventricular tachycardia.

### Electrocardiogram characteristics

All 55 patients exhibited a left superior axis deviation with a positive QRS complex in Leads I and aVL and a negative one in Leads III and aVF. In the LV-BIS group, an early precordial transition of ≤V2 was observed in all 28 patients. Among them, the QRS complex of a right bundle branch block (RBBB) pattern was observed in 21 patients (75.00%) and a left bundle branch block (LBBB) pattern in the remaining 7 (25.00%). In the patients in the RV-BIS group, a late precordial transition of ≥V3 with the QRS complex of an LBBB pattern was observed in all eight patients.

**Figure 1:**
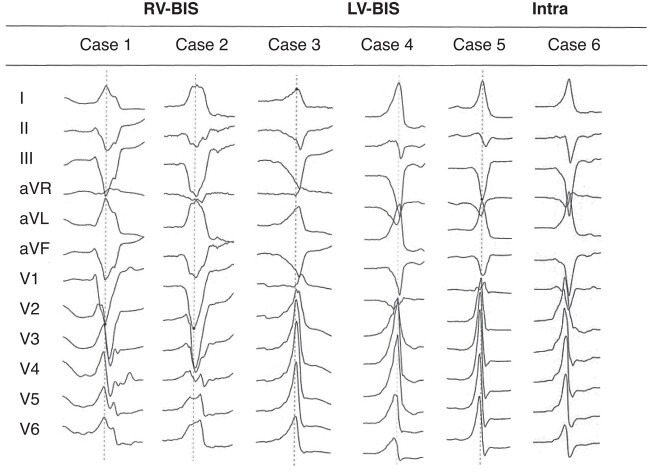
Representative ECG of BIS-VA. All patients exhibited a predominantly positive QRS complex in Leads I and avL, and a negative complex in inferior leads. VAs of RV-BIS featured with an LBBB pattern and a late precordial transition of ≥V3 (Cases 1 and 2), and VAs of LV-BIS exhibited as both RBBB (Case 3) and LBBB (Case 4) patterns with an early precordial transition of ≤V2, while the VAs of intramural origins also exhibited as both RBBB (Case 5) and LBBB (Case 6) patterns with an early precordial transition of ≤V2. BIS, basal inferior septum; ECG, electrocardiogram; Intra, intramural; LBBB, left bundle branch block; LV-BIS, left ventricle BIS; RBBB, right bundle branch block; RV-BIS, right ventricle BIS; VAs, ventricular arrhythmias.

In contrast, for the patients in the Intra group, the QRS complex varied: 4 patients (21.05%) presented an LBBB pattern with a precordial transition of >V2, 4 patients (21.05%) presented an RBBB pattern with an early precordial lead transition of ≤V2, and the remaining 11 patients (57.89%) exhibited an LBBB pattern with an early precordial transition of ≤V2. The typical ECG morphology in each group is presented in *Figure 1*.

The duration of QRS was comparable between the LV-BIS group and the Intra group but was longer in the RV-BIS group (Intra: 140.61 ± 10.49 ms; LV-BIS: 146.00 ± 19.89 ms; RV-BIS: 170.12 ± 11.70 ms, *P* < 0.001). An initial *r*/*R* wave in V1 was observed in 12 (42.86%), 3 (37.50%), and 6 (31.58%) in the LV-BIS, RV-BIS, and Intra groups (*P* = 0.807), respectively. But further measurements indicated that the initial *R*-wave amplitude was <0.2 mV in 5 of 6 patients in the Intra group (83.3%) and 9 of 12 patients (75.00%) in the LV-BIS group (*P* = 0.593). Besides, the maximal deflection index (MDI) was similar in the three groups (LV-BIS: 0.51 ± 0.05; RV-BIS: 0.48 ± 0.05; Intra: 0.49 ± 0.04, *P* = 0.09) as well as the rate of MDI >0.55 [LV-BIS: 6 (21.43%); RV-BIS: 1 (12.50%); Intra: 1 (5.26%), *P* = 0.336].

### Mapping findings

Because of aortic tortuosity, LV access was obtained via the transeptal approach in three patients in the Intra group and two patients in the LV-BIS group, and a retrograde approach was used in all the remaining cases.

**Figure 2:**
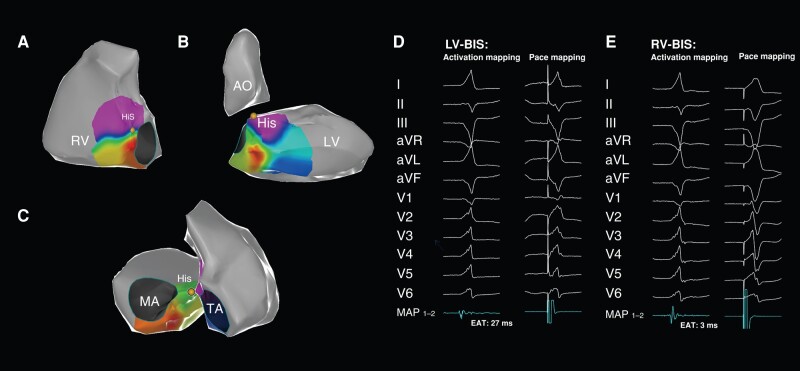
The mapping and ablation of VA from LV-BIS. Initial mapping along the TA found the EAS at the mid-septum (*A*), but the local activation time precedes QRS by only 3 ms and unmatched QRS during pace mapping (*E*). Further mapping along the MA identified the EAS at LV-BIS (*B*) with the local activation time >20 ms earlier than QRS and matched slightly different from QRS morphology during pace mapping (*D*), and ablation at this site successfully terminated the VA within 5 s. Combined maps showed an earlier local activation time at the EAS of LV-BIS (*C*). Of note, the QRS of V1 was featured with an LBBB pattern in this case, and even the success ablation target was on the left site. AO, aorta; EAT, earliest activation time; His, His bundle; LBBB, left bundle branch block; LV, left ventricle; LV-BIS, LV basal inferior septum; MA, mitral annulus; RV, right ventricle; RV-BIS, RV basal inferior septum; TA, tricuspid annulus; VA, ventricular arrhythmia.

**Figure 3:**
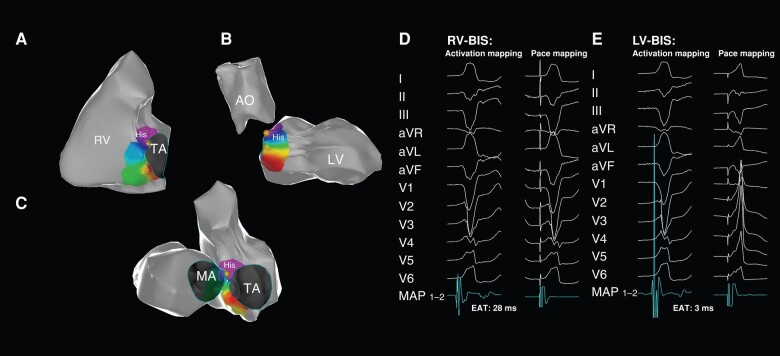
The mapping and ablation of VA from RV-BIS. VA from RV-BIS demonstrated significant EAT at the right-sided BIS with a perfectly matched QRS to the clinical VA (*A* and *D*). The later activation time was evident at the left-sided BIS with a poor-matched QRS during pace mapping (*B, C* and *E*). AO, aorta; EAT, earliest activation time; His, His bundle; LV, left ventricle; LV-BIS, LV basal inferior septum; MA, mitral annulus; RV, right ventricle; RV-BIS, RV basal inferior septum; TA, tricuspid annulus; VA, ventricular arrhythmia.

In the LV-BIS group, the local activation time at the left-sided BIS preceded QRS by 26.07 ± 1.96 ms, while the right-sided EAT preceded QRS only by 3.25 ± 1.56 ms (*P* < 0.001, *Figure 2*). In the RV-BIS group, the earliest local activation time on the right-sided BIS was 24.75 ± 1.91 ms earlier than that on a scalar QRS, which significantly preceded the opposite sites (3.75 ± 1.49 ms, *P* < 0.001, *Figure 3*).

**Figure 4:**
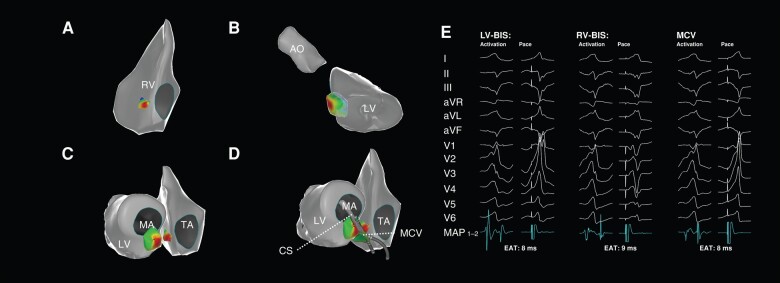
The mapping and ablation of VA of intramural origin. No adequate EAS was found at the two-sided BIS (*A, B* and *C*), and pacing at the breakouts produced the unmatched QRS to clinical VA (*E*). Further mapping was performed within the MCV and yielded similar results *(D*). Although a slightly earlier activation time was recorded in the ostium of the MCV, focal ablation on either side was futile, and extensive ablation was required to eliminate the VA. AO, aorta; BIS, basal inferior septum; CS, coronary sinus; EAT, earliest activation time; LV, left ventricle; MA, mitral annulus; MCV, middle cardiac vein; RV, right ventricle; TA, tricuspid annulus; VA, ventricular arrhythmia.

In the Intra group, all patients underwent epicardial MCV mapping. The EAS presented at the RV-BIS in nine patients (47.37%), at the LV-BIS in eight patients (42.11%), and in the MCV in two patients (10.53%). However, the earliest local activation time at the EAS preceded QRS only by 8.95 ± 2.19 ms. It was significantly delayed than that in the control group (LV-BIS group: 26.07 ± 1.96 vs. 8.95 ± 2.19 ms, *P* < 0.001; RV-BIS group: 24.75 ± 1.91 vs. 8.95 ± 2.19 ms, *P* < 0.001). The local activation times at the RV-BIS, LV-BIS, and MCV in the Intra group were comparable (7.79 ± 2.38 vs. 7.16 ± 2.59 vs. 6.26 ± 1.73 ms, respectively, *P* = 0.173, *Figure 4*). Moreover, the absolute activation time differences among these three sites were also comparable (RV-BIS vs. LV-BIS: 2.21 ± 1.08 ms; RV-BIS vs. MCV: 2.79 ± 1.32 ms; LV-BIS vs. MCV: 2.26 ± 1.73 ms, *P* = 0.384). No definite low-voltage area and abnormal late/diastolic potential were recorded around the BIS region in these patients, including those with sustained VT.

In the RV-BIS group, pacing from the EAS produced a matched QRS with clinical VAs in all patients, and pacing from the opposite side yielded a poorly matched QRS (*Figure 3*). In the LV-BIS group, pacing from the EAS produced a perfectly matched QRS (*Figure 2*) in 16 of 21 patients (76.19%) with the RBBB pattern and in 4 of 7 patients with the LBBB pattern (57.14%). While for patients in the Intra group, pacing either from the RV, LV-BIS, or MCV produced a QRS different from clinical VAs (*Figure 4*).

### Ablation outcomes

Initial ablation was directed at the EAS with an energy output of 35 W, with an irrigated saline of 17 mL/min. The VAs disappeared after an average of 3 ± 1 s of energy delivery in all patients in the RV-BIS and LV-BIS groups. Another energy delivery was continued at the same site.

**Figure 5:**
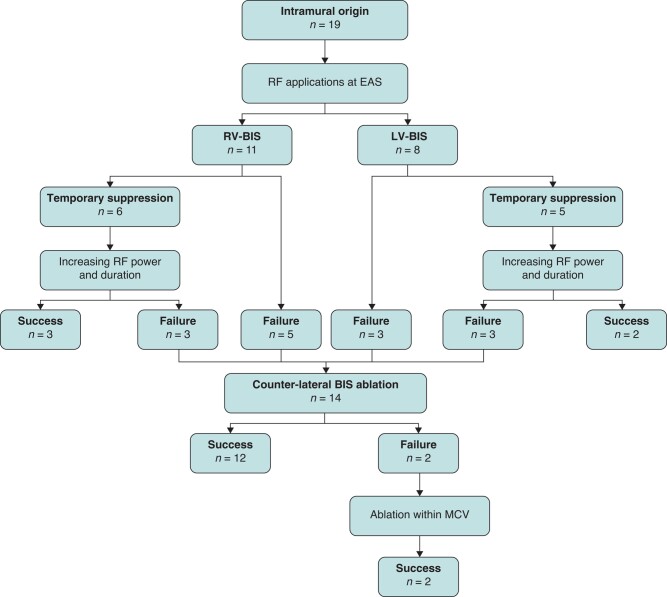
Flowchart of ablation outcome in the Intra group. BIS, basal inferior septum; EAS, earliest activation site; Intra, intramural; LV, left ventricle; MCV, middle cardiac vein; RF, radiofrequency; RV, right ventricle.

For patients with intramural origins, initial ablation targeted at the RV-BIS in 11 of 19 patients (57.89%), and the VAs were temporarily suppressed in 6 patients but recurred soon. By increasing the power settings and prolonging the ablation time on the same side, VAs were eliminated in three patients but remained unaffected in the remaining eight patients. For 8 of 19 patients (42.11%) whose initial ablation was at the LV-BIS, the VAs were temporarily suppressed in 5 patients. By increasing the power settings and prolonging the ablation time on the same side, VAs were eliminated in two patients but remained unchanged in the remaining six patients. Thereafter, ablation at the opposite BIS was performed in 14 patients who were refractory to the local radiofrequency applications. Finally, VAs were eliminated in 17 of 19 patients with an accumulative energy delivery of 187 ± 15 s after endocardial ablation. The last two patients achieved VA elimination through ablation within the MCV after endocardial ablation failure. The VA morphologies did not change throughout the procedure. Detailed ablation results are provided as a flowchart in *Figure 5*. Of note, a right atrial approach was attempted in 5 of 19 patients. But junctional rhythm was observed after about 10 s of radiofrequency applications. This approach was not followed thereafter. No junctional rhythm was observed in subsequent ablation. Overall, the rate of acute success was 100% across the three groups. The total ablation and procedural time were similar between the LV-BIS group and the RV-BIS group but were significantly longer in the Intra group. The procedural characteristics are summarized in *Table 2*.

**Table 2:** QRS characteristics and procedural data (*N* = 55)

	LV-BIS (*N* = 28)	RV-BIS (*N* = 8)	Intra (*N* = 19)	*P*-value
QRS duration, ms	140.61 ± 10.49	170.12 ± 11.70	146.00 ± 19.89	<0.001
Transition, *n* (%)				
≤V2	28 (100.00)	0	19 (100.00)	<0.001
>V2	0	8 (100)	0	
RBBB/LBBB	21/7	0/8	4/15	<0.001
MDI	0.51 ± 0.05	0.48 ± 0.05	0.48 ± 0.04	0.055
MDI > 0.55, *n* (%)	6 (21.43)	1 (12.50)	1 (5.26)	0.336
EAT, ms	26.07 ± 1.96	24.75 ± 1.91	8.95 ± 2.19	<0.001
EAT of LV-BIS, ms	26.07 ± 1.96	3.75 ± 1.49	7.16 ± 2.59	<0.001
EAT of RV-BIS, ms	3.25 ± 1.56	24.75 ± 1.91	7.79 ± 2.38	<0.001
EAT within MCV, ms	—	—	6.26 ± 1.73	—
Ablation time, s	139.14 ± 14.25	143.35 ± 15.06	189.06 ± 16.28	<0.001
Procedural time, min	65.32 ± 11.05	67.38 ± 10.16	92.47 ± 12.91	<0.001

BIS, basal inferior septum; EAT, earliest activation time; Intra, intramural; LBBB, left bundle branch block; LV, left ventricle; MCV, middle cardiac vein; MDI, maximal deflection index; RBBB, right bundle branch block; RV, right ventricle.

### Basal inferior septum reconstruction by intracardiac echocardiography

**Figure 6:**
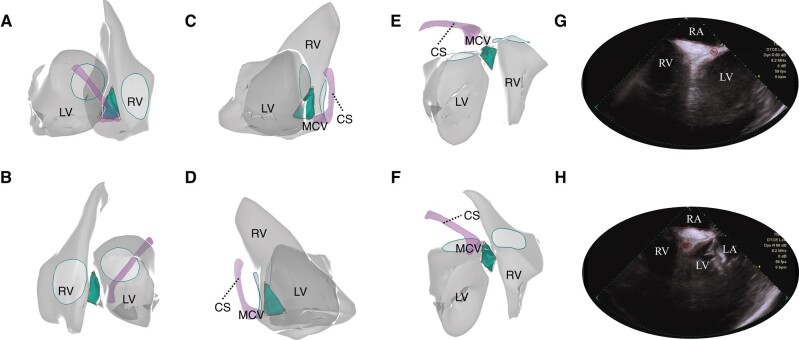
The anatomical configuration of the BIS region and adjacent structures under ICE view. The pyramid space (green) was a 3D structure displayed by ICE reconstruction. It was ‘sandwiched’ by the hinge line of the mitral and tricuspid valves and the inferior epicardial atrial-ventricular groove. When viewed from different projections, the pyramid served as the division of two-sided BIS with its vertices towards the upper septum and continuing with the muscular ventricular septum (*A–F*). Under the ICE fan, the pyramid space (red dashed line) was filled with adipose tissue with vessels passing through (red circles) (*G* and *H*). BIS, basal inferior septum; CS, coronary sinus; ICE, intracardiac echocardiography; LA, left atrial; LV, left ventricle; MCV, middle cardiac vein; RA, right atrial; RV, right ventricle.

Under the ICE view, the BIS was found to be the most basal and inferior process of the bi-ventricles, and between them was the pyramidal base, which was the thickest part of the ventricular septum, with an average thickness of 10.05 ± 1.49 mm at the transverse level (*Figure 6*). The pyramid averaged 22.09 ± 4.01 mm in height with a tapering thickness towards the upper septum.

### Complications and follow-up

No complications occurred in the patients during the peri-procedural period, and continuous ECG monitoring showed no evidence of VA recurrence before discharge. All patients off antiarrhythmic drugs were followed up for a median of 19.16 ± 4.17 months. Three patients in the Intra group experienced clinical VA recurrence with the same QRS morphology, and two of them received redo procedures. After focal ablation at the same site, VAs were eliminated after redo ablation. The condition of the other patient was well-controlled by antiarrhythmic drug therapy. Overall, the PVC burden decreased to 1.16 ± 0.47% at the last follow-up.

## Discussion

### Main findings

To the best of our knowledge, the current study was the first one to systematically investigate BIS-VAs of intramural origin. The following are the main findings of the study: (i) the pyramidal base is the broadest part of the septum and served as the division of the two-sided BIS; (ii) intramural VAs were relatively common in this region; (iii) the QRS morphology of the intramural VAs could be distinguished from the right-sided BIS-VAs by a shorter QRS duration and an early precordial lead transition of ≤V2, but it overlapped with the left-sided BIS-VAs; (iv) intracardiac mapping was essential for diagnosis; this was proved by recording the nearly simultaneous activation time and unmatched QRS during pace mapping; and (v) successful ablation could be achieved by performing ‘kissing’ ablations on the bilateral endocardial BIS as well as its epicardial aspect.

### Anatomy

The anatomy related to the BIS was initially described by cardiac surgeons who used the surgical approach to interrupt the septal accessary pathways and was examined later by a histological study to ascertain its relevance to catheter ablation.^7^ Based on autopsy hearts, Sánchez-Quintana *et al*.^8^ discovered that there is a pyramidal space bounded superiorly by the central fibrous body, anteriorly by the ventricular mass, and posteriorly by the convergence of the right and left atrial walls. The pyramidal space represents the confluence of all four cardiac chambers, and the hinge lines of MA and TA formed its leftward and rightward margins.^9^ In the presentg study, an offline reconstruction of this region was acquired via ICE sectors. The pyramidal space is exhibited as a 3D, tetrahedron structure: leftward, rightward, posterior, and inferior planes. Its anterior and posterior aspects are continued with the atrial and ventricular septum (consistent with the description of the ‘atrioventricular muscular septum’). Under the ICE view, this pyramid is featured with a broader base and tapering thickness towards the upper septum.

**Figure 7:**
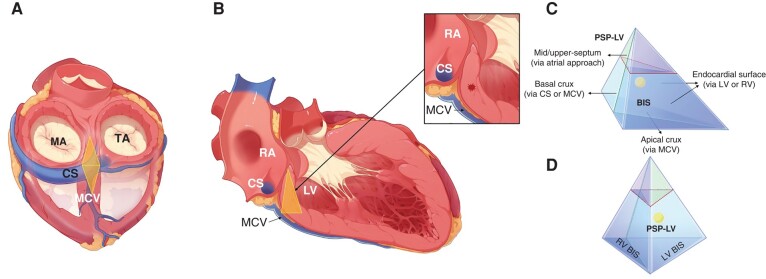
The anatomical relationship among the BIS, cardiac crux, and PSP-LV. The pyramidal space, outlined in the yellow area in (*A* and *B*), is the confluence of all four cardiac chambers. It is featured with a broader base and tapering thickness towards the upper septum, thus showing as a triangle surface towards different orientations. To elucidate the anatomical relationship among the BIS, cardiac crux, and PSP-LV, the pyramidal space is further simplified into a tetrahedron in (*C* and *D*). The BIS and PSP-LV are segmented from the pyramidal space by the red and green lines. As shown by the blue portion, the BIS is the pedestal of the pyramidal space and also inherits the endocardial and epicardial aspects of the pyramid. But, the PSP-LV represents the left-sided pyramidal space, extending from the anterior to the inferior septum. There is a prominent overlap between the PSP-LV and the BIS in the left basal process of the pyramidal space. At the same time, the crux cordis mainly represents the posterior and inferior epicardial aspects of the pyramid. The red asterisk in (*B*) and the yellow sphere indicate the intramural foci of the BIS-VAs settled deeply within the PSP-LV and BIS. BIS, basal inferior septum; CS, coronary sinus; LV, left ventricle; MA, mitral annulus; MCV, middle cardiac vein; PSP-LV, posterior–superior process of the LV; RA, right atrium; RV, right ventricle; TA, tricuspid annulus.

In previous studies, VAs from each of the pyramidal planes, such as the cardiac crux, bilateral BIS area, and posterior–superior process (PSP), have been described.^2–5,10–12^ The anatomy described in the present study partially overlaps with the anatomy described in previous studies. According to a description by Santangeli *et al*.,^12^ the inferior pyramidal area is the most basal inferior portion of the ventricle. This pyramidal structure has four sides, in which the ‘Crux cordis’ represents its epicardial aspect: the basal crux at its posterior side and the apical crux at its inferior side. The posterior–superior process of the left ventricle is the left-sided pyramid, including the mid-septum and the posterior septum. However, the BIS is the inferior portion of this pyramid, and the VAs described in this study are located within this structure, which has never been specifically addressed (*Figure 7*).

### Electrocardiogram characteristics

Previous studies focus on differentiating BIS-VAs from adjacent areas from different perspectives. Several ECG criteria have been proposed to distinguish VAs from the BIS, cardiac crux, and papillary muscles. Surface ECG criteria suggesting VA with a BIS origin include the following: the positive major component of the QRS complex on Leads I and aVL, a negative component in Leads III and aVF, and early *R* > *S* transition in precordial leads. Possibly because of their more anterior and rightward location, the current study found that the VAs of the right BIS exhibited a late precordial lead transition (>V2) and a longer QRS duration. However, considering the close adjacency of the right, the left, and the intramural locations of the BIS, a scalar ECG may not be accurate enough for differentiating BIS-VAs. As found in the current study, although the QRS morphology in Lead V1 manifested as an LBBB pattern in VAs arising from the right BIS, this was also frequently seen in VAs arising from the left-sided BIS or intramural origins. Meanwhile, there is a significant overlap of ECG presentations between the VAs from the left-sided BIS and the VAs with intramural origins in terms of QRS duration and precordial lead transition. The maximal deflection index proved to be an important clue for epicardial VAs, including the epicardial BIS region, namely the cardiac crux. The initial *R*-wave amplitude in V1 probably represents the distance between VAs foci and the endocardial surface within the ventricular septum. However, based on our observations, an MDI > 0.55 and *R*-wave amplitude in V1 were not reliable indicators for predicting BIS-VAs with intramural foci. Therefore, in those cases, detailed intracardiac mapping during the procedure was of importance.

### Electrophysiological characteristics of the ventricular arrhythmias from the intramural basal inferior septum

Intramural BIS-VAs were sporadically reported in previous case series. While no clear-cut diagnostic criteria exist, VAs with intramural origins are often considered only after extensive mapping and failed ablation. Bogun’s group^6,10^ proposed the use of dual-site pacing at the adjacent breakouts. They found that if the averaged pacing ECGs were closer to clinical VA than single-site pacing, an intramural origin was suggested. Their group also reported a cut-off value of ≤8 ms between the breakouts at adjacent anatomic areas to differentiate intramural VAs from non-intramural VAs with a specificity of 92% in the absence of a matching pace map. However, most of their study cases were related to the ventricular outflow tract. Whether the criteria are suitable for BIS-VAs remains unknown.

As proposed by Larsen *et al*.,^11^ the mapping of multiple chambers (including counterpart BIS and epicardium) is of importance for locating the origin of BIS-VAs. The current study found that the activation time at the counterpart breakouts averaged <5 ms in difference, and the pacing-produced QRS is poor-fitted to the clinical QRS complex in intramural BIS-VAs. However, in the control group with local endocardial origins, the absolute activation time at the EAS was usually 20 ms preceding QRS, and the activation time difference of the bilateral endocardial breakouts was over 20 ms. Therefore, if no absolute EAS is identified on a certain side or/and the opposite side, as well as within the MCV, an intramural origin should be suspected. Certainly, mapping through the coronary venous system would further define the intramural BIS-VAs by recording the not early activation time on the inferior BIS. But a comparable activation time within the MCV would exclude the epicardial crux VAs. Intramural origin was further suspected by pacing mapping. Pacing from the EAS on either side of the pyramidal BIS could not produce a perfect match with the clinical VAs in intramural VAs, also indicating its mid-myocardial origin. Therefore, the electrophysiological characteristics, especially the activation time and pace mapping at the adjacent breakouts, would provide useful clues.

### Ablations of basal inferior septum ventricular arrhythmias with intramural origins

In the current series, a stepwise ablation strategy was applied to treat intramural BIS-VAs, which yielded promising acute-term and long-term outcomes. Based on our observation, compared with endocardial BIS-VAs, a few cases with intramural foci were amendable to single endocardial site ablation. The majority required a ‘kissing’ ablation at the counterlateral breakouts. This finding is consistent with that of a previous study, in which about half of the patients required ablation from at least two adjacent sites.^11^ In addition, in two patients, successful ablations were achieved by ablations within the MCV after failed ablations at the bilateral endocardial aspect. Whether this success was due to accumulative effects or just local effects within the MCV remains unclear. In one recent study, successful ablation was achieved by ablation from the atrial side.^12,13^ However, an atrioventricular block tended to occur because a rapid junctional rhythm was frequently observed when attempted ablations at this site were observed in some patients in the present study.

### The possible mechanism of ventricular arrhythmia genesis

Different from the previous series, VAs with intramural origins were commonly seen in the pyramidal space, accounting for ∼34.5% of BIS-VAs in our study. Ventricular arrhythmias have a predilection to originate from embryologic raphe structures, such as a junction between a vessel and the myocardium or between an annulus and the myocardium. The pyramidal space is not only a muscular structure but rather an area encompassing the coronary sinus, the posterior descending branch of the right coronary artery, and epicardial fat.^4^ Inoue and Becker^9^ reported that among 21 randomly selected and basically normal hearts obtained from autopsies, 13 showed posterior extensions of the atria-ventricular node on both the left and the right sides, seven showed a rightward posterior extension only (run close to the TA), and only one heart showed a single leftward extension (run close to the MA). Besides, some cells with nodal type activation potentials were found in this area by microelectrode recordings.^14^ Therefore, not only would pyramidal VAs arise from the musculature itself, but these intramural structures would also provide arrhythmogenic substrates and result in different preferential activation exits between the bilateral pyramids. However, in contrast to the LV summit, which is thicker with more complicated arrangements, the average thickness of the pyramidal base is usually around 10 mm as measured by ICE in the present study; therefore, it is possible to create effective lesions in the intramural part by the accumulative effect via ‘kissing’ ablations.

Other alternative approaches, such as half-normal saline, needle-tip ablation, ethanol ablation, and bipolar ablation, have been proposed to target intramural VAs.^1,15–19^ However, needle ablation was not commercially available. Ethanol ablation was not considered because it was found that no suitable blood vessel reached this part or there was a concern about atrial-ventricular conduction disturbance. Bipolar ablation is essentially equal to an alternatively unipolar ablation at both sides because both modalities depend on heat conduction to create lesions. Enhancing lesions with half-normal saline irrigation is a delicate process, and therefore, it is not routinely used.

### Limitations

This study has several limitations. First, magnetic resonance imaging was not routinely performed in all patients, and therefore, there is the possibility that early-stage arrhythmogenic right and/or LV cardiomyopathy might be overlooked. However, no definite scar was identified during intracardiac mapping. And the finding of the magnetic resonance imaging did not affect the mapping and ablation strategies adopted in this study. Secondly, our study did not reveal the precise mechanism of VAs from the BIS, which warrants further study. Thirdly, epicardial mapping via the subxiphoid approach was not performed, which would provide more valuable information on intramural BIS-VAs. Fourthly, the acute success rate of 100% may not be reliable after only 30 min of waiting, because some recurrent VA could be documented after a longer observation, considering that some patients experienced recurrence even after acute success was achieved. Finally, this is a single-centre study, in which the mapping and ablation strategies might be operator-dependent.

## Conclusions

Ventricular arrhythmias with an intramural origin are common within the BIS region. A scalar ECG provides limited clues, but intracardiac mapping is important for distinguishing the VAs of intramural origins from the other VAs by comparing the local activation time and pacing mapping results. In patients with such VAs, ‘kissing’ ablation on the counterpart sides of BIS as well as within the MCV was usually effective.

## Data Availability

Raw data are available upon reasonable request to the corresponding author.
